# Roles of tumor necrosis factor alpha gene polymorphisms, tumor necrosis factor alpha level in aqueous humor, and the risks of open angle glaucoma: A meta-analysis

**Published:** 2013-02-27

**Authors:** Xiangyang Xin, Lili Gao, Tong Wu, Fengyuan Sun

**Affiliations:** 1 First Center Clinical College, Tianjin Medical University, Tianjin, P. R. China; 2 Department of Ophthalmology, Inner Mongolia Baogang Hospital, Inner Mongolia, P. R. China; 3 Department of Ophthalmology, Tianjin First Center Hospital, Tianjin Medical University, Tianjin, P. R. China

## Abstract

**Purpose:**

This meta-analysis was performed to clarify the association between tumor necrosis factor alpha (*TNF-α*) gene polymorphisms and open angle glaucoma (OAG) risks, and the association between the TNF-α level in aqueous humor (AH) and the risks of glaucoma.

**Methods:**

A computerized literature search was performed for the relevant available studies from three databases including PubMed, ISI Web of Science, and Embase. The fixed or random effect model was selected based on the heterogeneity test using the Q test and the *I^2^* statistic. The associations between *TNF-α* gene polymorphisms and OAG risks were estimated by calculating pooled odds ratios (ORs) and the 95% confidence interval (CI), while a pooled standardized mean difference with 95% CI was used for the comparison of TNF-α levels in AH between patients with OAG and controls. Publication bias was estimated using Begg’s funnel plots and Egger’s regression test.

**Results:**

A total of 14 (1,182 cases and 3,003 controls), five (808 cases and 1,039 controls), three (645 cases and 666 controls), and three studies (404 cases and 625 controls) were finally included in the analyses for the associations between *TNF-α* −308G/A, −857C/T, −863C/A, and −238G/A polymorphisms and the risks of OAG, respectively. The combined results showed that the *TNF-α* −308G/A gene polymorphism was significantly associated with risks of high-tension glaucoma (A versus G: OR=1.660, 95% CI=1.033–2.667; AA/AG versus GG: OR=1.713, 95% CI=1.10–2.651), but not with normal tension glaucoma or exfoliation glaucoma. Ethnicity-stratified analysis revealed that a significant association also existed in Asians (A versus G: OR=1.947, 95% CI=1.097–3.456; AA/AG versus GG: OR=1.949, 95% CI=1.140–3.332). None of the other polymorphisms was significantly associated with OAG risks. Furthermore, the pooled results of six studies showed that the TNF-α levels in the AH of patients with OAG was higher than that of the control subjects (standardized mean difference=0.517, 95% CI=0.207–0.826, p=0.001). Probability of publication bias was low across all comparisons illustrated by the funnel plots and Egger’s test.

**Conclusions:**

This meta-analysis suggests that patients with OAG may have higher TNF-α levels compared with the control subjects, and the *TNF-α* −308G/A polymorphism is significantly associated with the risks of high-tension glaucoma. Since potential confounders could not be ruled out completely, further studies are needed to confirm these results.

## Introduction

Glaucoma, characterized by optic neuropathy and a progressive loss of the retinal ganglion cells (RGCs), is the second leading cause of blindness worldwide [1]. Glaucoma is classified into primary and secondary glaucoma according to the etiology and aqueous humor dynamics. Based on the anatomy of the anterior chamber, primary glaucoma is further classified as primary open angle glaucoma (POAG) and primary angle closure glaucoma (PACG). High-tension glaucoma (HTG) and normal tension glaucoma (NTG) are types of POAG, while exfoliation glaucoma (EXG) is a type of secondary glaucoma. Open angle glaucoma (OAG), including HTG, NTG, and EXG, is the most common form of glaucomas, accounting for about 90% of glaucoma cases in the United States [2]. The etiology of OAG is complex, and not fully understood at present. Although elevated intraocular pressure (IOP) has been recognized as the major risk factor for OAG, elevated IOP is not a diagnostic factor [2]. Furthermore, currently available drugs primarily aim to lower the IOP; however, the disease progression may continue despite significant IOP reduction [3]. Apparently, other factors other than IOP may also play important roles in the pathogenesis of glaucomatous optic neuropathy.

Tumor necrosis factor-alpha (TNF-α) is a cytokine that belongs to the TNF superfamily of 19 different protein ligands [4]. TNF-α is a proinflammatory cytokine with multiple functions in the immune response. Accumulating studies strongly support the involvement of TNF-α in the etiology of glaucoma [3,5]. Ischemic or pressure-loaded glial cells could produce TNF-α, which results in oligodendrocyte death and the subsequent apoptosis of RGCs [6]. The expression of TNF-α and TNF-α receptor-1 (TNF-R1) was upregulated in the retina and the optic nerve head, and the expression of TNF-α and TNF-R1 appeared to parallel the progression of optic nerve degeneration [6-8]. Furthermore, TNF-α was upregulated as a consequence of increasing IOP, and exogenous TNF-α could also lead to loss of oligodendrocytes and a delayed loss of RGCs [9]. The same study also demonstrated that functional blockade of TNF-α with an anti-TNF-α blocking antibody or deletion of the gene encoding TNF-α in a genetically altered mouse model prevented ocular hypertension (OH)-induced oligodendrocyte degeneration and the secondary loss of RGCs [9]. This research suggests that TNF-α may play a key role in glaucomatous neurodegeneration.

The human TNF-α gene is located within the highly polymorphic major histocompatibility complex class III region on chromosome 6p21.3 [10]. Several functional polymorphisms in the promoter region of the TNF-α gene have been identified, and have been related to the risks of glaucoma. The intensively studied polymorphism is characterized by a G to A substitution at position −308 (−308G/A, rs1800629), which could increase transcription six- to sevenfold [11]. Other polymorphisms including −238G/A (rs361525), −863C/A (rs645836), and −857C/T (rs1799724) have also been studied. However, the results have been highly inconsistent. Some studies suggested that the *TNF-α* −308 A allele was significantly associated with the increased risk of OAG [12-15], while other studies showed no significant association [16-18], and even reduced risks of OAG [19,20]. Furthermore, to investigate the roles of TNF-α and risks of glaucoma, several recent studies detected the TNF-α level in the aqueous humor (AH) in patients with glaucoma and the control subjects. Similarly, some studies showed that the TNF-α concentrations in AH and/or the ratio of TNF-α positive AH samples in patients with glaucoma was higher than those in controls [21,22], which was disclaimed by other studies [23].

In the current study, we performed a meta-analysis of all eligible studies, to provide a more accurate estimate of the association of the *TNF-α* gene (−308G/A, −238G/A, −863C/A, and −857C/T) polymorphisms and the risks of OAG. We also performed a meta-analysis to compare the TNF-α level in AH between patients with glaucoma and the control subjects.

## Methods

### Literature and search strategy

A computerized literature search was performed for the relevant available studies from three databases including PubMed, ISI Web of Science, and Embase. The search strategy for identifying the relevant studies involved combinations of the following keywords: “tumor necrosis factor-α” or “TNF-α” and “glaucoma.” The reference lists of review articles, clinical trials, and meta-analyses were also hand-searched to collect other relevant studies. If more than one article was published using the same case series, only the study with the largest sample size was selected. The literature search was updated on August 30, 2012.

The inclusion criteria were as follows: (1) evaluating the associations between *TNF-α* (−308G/A, −238G/A, −863C/A, or −857C/T) polymorphisms and the risk of OAG or comparing the TNF-α level in AH of patients with glaucoma and that of the control subjects, (2) case-control design, and (3) gene polymorphisms studies must provide sufficient data for calculating the odds ratio (OR) and the corresponding 95% confidence interval (95% CI). All identified studies were carefully reviewed independently by two investigators to determine whether an individual study was eligible for inclusion in this meta-analysis

### Data extraction

Data were extracted independently by two investigators who reached a consensus on all of the items. The following information was extracted from each study: (1) name of the first author, (2) year of publication, (3) country of origin, (4) ethnicity of the study population, (5) number of cases and controls, (6) gender and age of enrolled subjects, and (7) number of genotypes in cases and controls.

### Statistical analysis

Genotypic frequency for the *TNF-α* gene polymorphisms was tested for deviation from Hardy–Weinberg equilibrium (HWE) in the control subjects using the chi-square goodness of fit. The associations between the *TNF-α* (−308G/A, −238G/A, −863C/A, and −857C/T) polymorphisms and OAG risks were estimated by calculating pooled ORs and 95% CI. The significance of the pooled effect size was determined with the *Z* test. Heterogeneity among studies was assessed using the Q test as well as the *I^2^* statistic, which was documented for the percentage of the observed between-study variability due to heterogeneity rather than chance [24]. A significant Q-statistic (p<0.10) indicated heterogeneity across studies. The DerSimonian and Laird random effect model (REM) was used if heterogeneity existed; otherwise, the Mantel-Haenszel fixed effect model (FEM) was used [24]. Subgroup analyses were stratified by ethnicity and the type of OAG. Sensitivity analysis was conducted by removing an individual study each time to check whether a single study could bias the overall estimate [25]. Begg’s funnel plots and Egger’s regression test were performed to assess the potential publication bias [26].

A pooled standardized mean difference (SMD), together with 95% CI, was used to compare the TNF*-α* level in AH between the patients with OAG and the controls. The SMD was chosen because the TNF*-*α concentrations were measured using different methods in the included studies. The heterogeneity test was also performed by using the Q test and the *I^2^* statistic as performed in other studies [27,28]. In cases of no statistical heterogeneity, the FEM was used for SMD meta-analysis; otherwise, the REM was chosen as the appropriate choice. Begg’s funnel plots and Egger’s linear regression were used to assess evidence for publication bias.

All statistical analysis was performed using STATA version 11 (StataCorp LP, College Station, TX). Two-sided p values less than 0.05 were considered statistically significant.

## Results

### Characteristics of studies included for the association between tumor necrosis factor alpha gene polymorphisms and open angle glaucoma risks

Eleven studies investigating the association between *TNF-α* gene polymorphisms and OAG risks were retrieved. Among these studies, Fan et al. recruited three forms of patients with OAG [19], while Razeghinejad et al. and Funayama et al. investigated two forms of OAG [13,18]. Three studies investigated the association of OAG risks with *TNF-α* (−308G/A) and *TNF-α* (−238G/A) [16,17,29], one study investigated the association of OAG with *TNF-α* (−308G/A) and *TNF-α* (−857C/T) [19], and one study investigated the association of OAG with three single nucleotide polymorphisms, *TNF-α* (−308G/A), *TNF-α* (−857C/T), and *TNF-α* (−863C/A) [18]. All other studies investigated a single nucleotide polymorphism in one form of OAG [12,14,15,20,30]. Finally, 14 (1,182 cases and 3,003 controls), five (808 cases and 1,039 controls), three (645 cases and 666 controls), and three studies (404 cases and 625 controls) were included in the analyses for the association between *TNF-α* −308G/A, −857C/T, −863C/A, and −238G/A polymorphisms and risks of OAG, respectively. The studies used blood samples for DNA extraction, while polymerase chain reaction-restriction fragments length polymorphism, TaqMan, or DNA sequencing methods were used for genotyping. Genotype distribution in control groups were in HWE in most of the studies except two studies for the *TNF-α* (−308G/A) polymorphism [14,19]. Details of the studies are shown in Table 1.

**Table 1:** Characteristics of individual studies for associations between *TNF-α* polymorphisms and risks of open angle glaucoma

Authors	Year	Country	Type	Gender	Age^a^	Genotypes distribution								*P*_HWE_^c^
						Case ^b^				control ^b^				
						11	12	22		11	12	22		
***-308G/A (rs1800629)***														
Bozkurt	2012	Turkey	HTG	40.7/38.3	65.1/66.2	66	19	1		171	21	1		0.686
Al-Dabbagh	2011	Saudi Arabia	HTG	47/50	58/55	59	68	8		110	76	14		0.860
Fan	2010	China	HTG	65.5/59.7	62.8/69.8	226	25	1		167	29	5		0.012
Fan	2010	China	NTG	54/59.7	63.2/69.8	87	12	0		167	29	5		0.012
Fan	2010	China	HTG/NTG	66/59.7	21.3/69.8	37	9	0		167	29	5		0.012
Razeghinejad	2009	Iran	HTG	na	na	57	13	0		190	10	0		0.717
Razeghinejad	2009	Iran	EXG	na	na	48	10	1		190	10	0		0.717
Mossbock	2009	Austria	EXG	42.2/-	76.1/75.3	152	48	4		151	51	2		0.305
Khan	2009	Pakistan	EXG	67/64	45.3/44.2	53	39	30		110	13	3		0.004
Tekeli	2008	Turkey	EXG	55.5/51.8	66.0/64.2	103	7	0		92	18	0		0.350
Mossbock	2006	Austria	HTG	42.1/42.1	72.3/72.7	79	35	0		161	61	6		0.938
Funayama	2004	Japan	HTG	56.7/42.2	65.1/70.6	192	2	0		212	6	0		0.836
Funayama	2004	Japan	NTG	44.7/42.2	60.3/70.6	211	6	0		212	6	0		0.836
Lin	2003	China	HTG	50/53.4	55/50	28	13	19		66	30	7		0177

***-857C/T (rs1799724)***														
Fan	2010	China	HTG	65.5/59.7	62.8/69.8	189	58	5		159	39	3		0.732
Fan	2010	China	NTG	54/59.7	63.2/69.8	80	19	0		159	39	3		0.732
Fan	2010	China	HTG/NTG	66/59.7	21.3/69.8	35	11	0		159	39	3		0.732
Funayama	2004	Japan	HTG	56.7/42.2	65.1/70.6	135	49	10		144	69	5		0.324
Funayama	2004	Japan	NTG	44.7/42.2	60.3/70.6	148	64	5		144	69	5		0.324

***-863C/A (rs645836)***														
Wang	2012	China	HTG	52/53	67/67	148	70	16		120	84	26		0.062
Funayama	2004	Japan	HTG	56.7/42.2	65.1/70.6	141	46	7		161	56	1		0.093
Funayama	2004	Japan	NTG	44.7/42.2	60.3/70.6	159	55	3		161	56	1		0.093

***-238G/A (rs361525)***														
Bozkurt	2012	Turkey	HTG	40.7/38.3	65.1/66.2	79	7	0		180	13	0		0.628
Mossbock	2009	Austria	EXG	42.2/-	76.1/75.3	182	21	1		189	15	0		0.586
Mossbock	2006	Austria	HTG	42.1/42.1	72.3/72.7	107	7	0		205	23	0		0.423

^a^ the mean age of case and control; ^b^ 1 represent the common allele, while 2 represents the minor allele. ^c^ *p* for Hardy–Weinberg equilibrium test in controls; “na“ means that the data were not available; Abbreviations: HTG, high-tension glaucoma; NTG, normal-tension glaucoma; EXG, exfoliative glaucoma.

### Quantitative data synthesis

**Figure 1:**
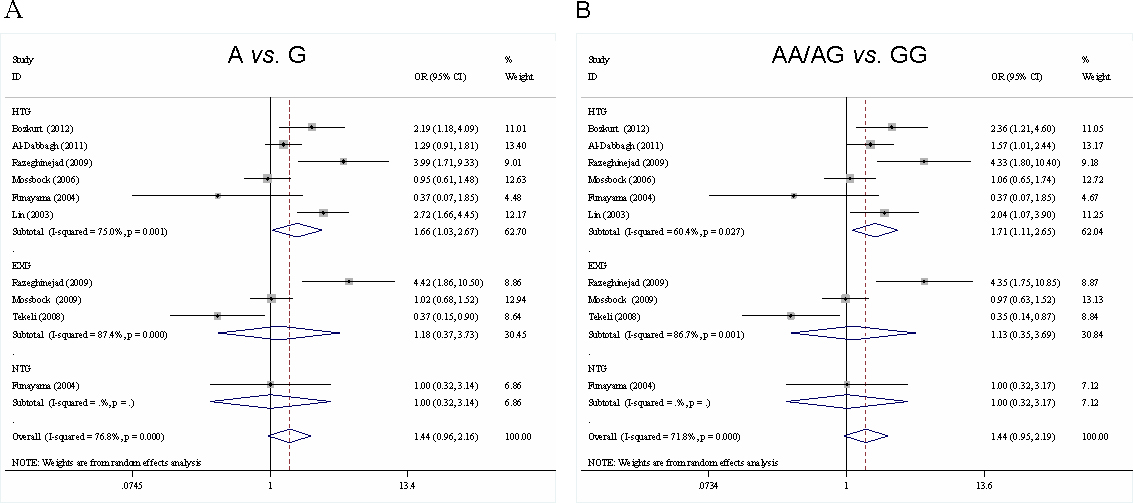
Meta-analysis for the *TNF-α* −308G/A (rs1800629) polymorphism and risks of open angle glaucoma. Each study is shown by a point estimate of the effect size (OR; size inversely proportional to its variance) and its 95% confidence interval (95% CI; horizontal lines). The white diamond denotes the pooled OR. HTG, high-tension glaucoma; NTG, normal tension glaucoma; EXG, exfoliative glaucoma. Meta-analysis indicated a statistical significant association between the *TNF-α* −308G/A polymorphism with the risks of HTG, but not with the risks of NTG or EXG.

Results of pooled analysis on the associations between *TNF-α* −308G/A polymorphisms and OAG risks are shown in Table 2. As shown in Table 2, when all the studies were included in the analysis, no significant association was observed between the *TNF-α* −308G/A polymorphism and the risk of OAG (A versus G: OR=1.379, 95% CI=0.877–2.170; AA/AG versus GG: OR=1.421, 95% CI=0.907–2.226). Subgroup analysis stratified by type of OAG and ethnicity further confirmed the null association except the homozygous genotype comparison for EXG. However, when the two studies in which the genotype distribution in the control groups deviated from HWE were excluded [14,19], a significant association was observed between *TNF-α* −308G/A polymorphisms and the risks of HTG (A versus G: OR=1.660, 95% CI=1.033–2.667; AA/AG versus GG: OR=1.713, 95% CI=1.10–2.651), but not with NTG (A versus G: OR=1.005, 95% CI=0.321–3.140; AA/AG versus GG: OR=1.005, 95% CI=0.319–3.165) or EXG (A versus G: OR=1.181, 95% CI=0.373–3.732; AA/AG versus GG: OR=1.130, 95% CI=0.346–3.689). Furthermore, ethnicity-stratified analysis revealed that a significant association also existed in Asians (A versus G: OR=1.947, 95% CI=1.097–3.456; AA/AG versus GG: OR=1.949, 95% CI=1.140–3.332) (Table 2 and Figure 1).

**Table 2:** Meta-analysis for the association between *TNF-α* −308G/A (rs1800629) polymorphisms and risks of open angle glaucoma

Contrast		All relevant articles were included (n=14)					Articles deviated for HWE were excluded (n=10)^a^				
		OR	95%CI	M ^b^	*I*^2^(%)	P *value* ^c^	OR	95%CI	M ^b^	*I*^2^(%)	P *value*^c^
A versus G	HTG	1.374	0.825–2.290	R	82.6	0.000	1.660	(1.033–2.667)*	R	75.0	0.001
	NTG	0.680	0.384–1.204	F	0.0	0.000	1.005	0.321–3.140	F	–	–
	EXG	1.957	0.515–7.442	R	94.6	0.445	1.181	0.373–3.732	R	87.4	0.000
	Caucasian	1.005	0.596–1.695	R	72.5	0.012	1.005	0.596–1.695	R	72.5	0.012
	Asian	1.591	0.853–2.966	R	89.4	0.000	1.947	(1.097–3.456)*	R	73.3	0.002
	ALL	1.379	0.877–2.170	R	87.4	0.000	1.440	0.958–2.164	R	76.8	0.000
AA versus GG	HTG	1.047	0.245–4.467	R	75.1	0.003	1.688	0.404–7.055	R	71.0	0.016
	NTG	0.174	0.010–3.184	F	–	–	–	–	–	–	—
	EXG	7.918	(1.473–42.558)*	R	58.5	0.090	2.896	0.653–12.841	F	0.0	0.338
	Caucasian	0.896	0.296–2.714	F	28.4	0.248	0.896	0.296–2.714	F	28.4	0.248
	Asian	1.793	0.446–7.211	R	80.2	0.000	3.277	0.716–14.990	R	74.1	0.021
	ALL	1.585	0.525–4.787	R	73.6	0.000	2.133	0.745–6.105	R	56.1	0.044
GA versus GG	HTG	1.349	0.843–2.159	R	70.2	0.003	1.580	(1.006–2.477)*	R	59.0	0.032
	NTG	0.848	0.461–1.557	F	0.0	0.734	1.005	0.319–3.165	F	–	–
	EXG	1.684	0.506–5.608	R	91.1	0.000	1.075	0.344–3.360	R	85.3	0.001
	Caucasian	1.028	0.571–1.849	R	73.5	0.010	1.028	0.571–1.849	R	73.5	0.010
	Asian	1.549	0.914–2.625	R	78.3	0.000	1.688	0.941–3.030	R	62.9	0.019
	ALL	1.359	0.916–2.018	R	76.9	0.000	1.341	0.883–2.036	R	69.8	0.000
AA/GA versus GG	HTG	1.421	0.865–2.334	R	75.6	0.000	1.713	(1.107–2.651)*	R	60.4	0.027
	NTG	0.752	0.414–1.367	F	0.0	0.567	1.005	0.319–3.165	F	–	–
	EXG	1.921	0.488–7.560	R	93.7	0.000	1.130	0.346–3.689	F	86.7	0.001
	Caucasian	1.018	0.571–1.815	R	73.5	0.010	1.018	0.571–1.815	R	73.5	0.010
	Asian	1.652	0.897–3.041	R	85.5	0.000	1.949	(1.140–3.332)*	R	60.1	0.028
	ALL	1.421	0.907–2.226	R	83.6	0.000	1.440	0.949–2.815	R	71.8	0.000

^a^ studies by Fan et al., and Khan et al.. were excluded as they were deviated from Hardy–Weinberg equilibrium; ^b^ M, model for meta-analysis; F, fixed effect model; R, random effect model; ^c^ p value for heterogeneity based on Q test; HTG, high-tension glaucoma; NTG, normal-tension glaucoma; EXG, exfoliative glaucoma; *p<0.05.

The pooled results on the associations between the *TNF-α* (−238G/A, −863C/A, and −857C/T) polymorphisms and the OAG risks are shown in Table 3. The combined results showed that none of these gene polymorphisms was significantly associated with the risks of OAG (for −857C/T, T versus C, OR=1.055, 95% CI=0.931 −1.216; TT/CT versus CC, OR=0.984, 95% CI=0.794–1.219; for −863C/A, A versus C, OR=0.928, 95% CI=0.636–1.353; AA/AC versus CC, OR=0.871, 95% CI=0.618–1.227; for −238G/A, OR=1.088, 95% CI=0.691–1.941; AA/AG versus GG, OR=1.076, 95% CI=0.681–1.701). Due to the limited studies available, we did not conduct a sub-group analysis for the *TNF-α* (−238G/A, −863C/A, −857C/T) polymorphisms.

**Table 3:** Meta-analysis for the association between *TNF-α* −857C/T, −863C/A, and −238G/A polymorphisms and the risks of open angle glaucoma

Contrast	Number of studies	OR	95%CI	M ^a^	*I*^2^(%)	P *value* ^b^
***-857C/T (rs1799724)***						
T versus C	5	1.055	0.831–1.216	F	0.0	0.798
TT versus CC	5	1.261	0.659–2.14	F	0.0	0.694
CT versus CC	5	0.967	0.775–1.206	F	0.0	0.548
TT/CT versus CC	5	0.984	0.794–1.219	F	0.0	0.705

***-863C/A (rs645836)***						
A versus C	3	0.928	0.636–1.353	R	70.3	0.035
AA versus CC	3	1.855	0.273–12.623	R	75.0	0.018
AC versus CC	3	0.844	0.660–1.078	F	0.0	0.375
AA/AC versus CC	3	0.871	0.618–1.227	F	52.1	0.124

***-238G/A (rs361525)*** ^c^						
A versus G	3	1.088	0.696–1.941	F	34.7	0.216
AA/GA versus GG	3	1.076	0.681–1.701	F	31.7	0.232

^a^ M, model for meta-analysis; F, fixed effect model; R, random effect model; ^b^ p value for heterogeneity based on Q test. ^c^ for *−238G/A (rs361525)* polymorphism, we only made the comparison of “A vs. G” and “AA/GA vs. GG” due to the smaller frequency of genotype AA.

### Sensitivity analysis and publication bias

**Figure 2:**
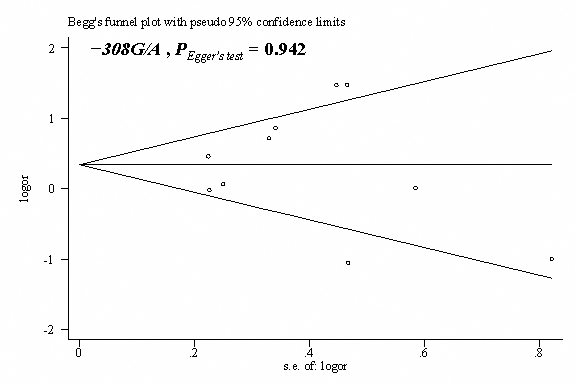
Begg’s funnel plot with the Egger’s test for publication bias between *TNF-α* −308G/A (rs1800629) polymorphisms and risks of open angle glaucoma. The horizontal line in the funnel plot indicates the fixed-effects summary estimate, whereas the diagonal lines pseudo-95% CI limits about the effect estimate. In the absence of publication bias, studies are distributed symmetrically above and below the horizontal line.

Sensitivity analysis was performed by sequential omission of individual studies in every comparison, and the data showed that no study significantly influenced the pooled effects by omitting any study. After studies that deviated from HWE in controls were excluded, no other studies were found to significantly influence the pooled effects in each genetic model. Begg’s funnel plots were generated to assess publication bias. Egger’s test was performed to statistically evaluate funnel plot symmetry. The results suggested no publication bias for the association of the *TNF-α* (−308G/A, −238G/A, −863C/A, and −857C/T) polymorphisms and the OAG risks (*P*_Egger test_ *>*0.05; Figure 2).

### Pooled results of tumor necrosis factor alpha level in aqueous humor of patients with glaucoma and the control subjects

Five studies in which the TNF*-*α levels in AH between patients with glaucoma and control subjects were compared were retrieved. Enzyme-linked immunosorbent assay, singleplex bead immunoassay analysis, multiplex bead immunoassay, and microparticle-based immunoassays were used to measure the TNF*-*α concentration in the AH in different studies. The TNF*-*α level was below the detection limit in one study [31], while Takai et al. recruited two forms of patients with OAG [23], and Sawada et al. enrolled three forms of patients with OAG [32]. Some studies provided the mean and standard deviation of TNF-α levels in patients with OAG and controls, while other studies reported the TNF-α positive and negative AH samples in patients with OAG and controls. Details of the retrieved studies are shown in Table 4.

**Table 4:** Summary of the studies for the TNF-α level in the aqueous humor between glaucoma patients and the control subjects

Authors	Year	Country	Type ^a^	TNF-α level (pg/ ml)							TNF-α positive samples (P/N) ^c^	
				Case ^b^				Control ^b^				
				n	m	SD		n	m	sd	Case	Control
Takai	2012	Japan	POAG	19	1.6	1.2		21	1.5	0.7		
Takai	2012	Japan	EXG	20	2.3	1.7		21	1.5	0.7		
Balaiya	2011	USA	POAG	32	2.72	1.5		32	1.59	0.46		
Sawada	2010	Japan	POAG	4	14.1	12.8		4	17.9	13		
Sawada	2010	Japan	NTG	3	23.9	9.6		4	17.9	13		
Sawada	2010	Japan	EXG	8	12.8	13		4	17.9	13		
Chua	2012	China	POAG								24/2	16/7
Takai	2012	Japan	POAG								19/1	21/0
Takai	2012	Japan	EXG								20/3	21/0
Sawada	2010	Japan	POAG								4/25	4/75
Sawada	2010	Japan	NTG								3/25	4/75
Sawada	2010	Japan	EXG								8/19	4/75

This table summarized the published studies investigating the TNF-α level in the aqueous humor between glaucoma patients and the control subjects. In some studies, the TNF-α level were shown as the mean ± SD, while in some other studies the TNF-α levels were shown as the percentage of the TNF-α positive samples. ^a^ POAG, primary open angle glaucoma; NTG, normal-tension glaucoma; EXG, exfoliative glaucoma; ^b^ n, m, SD, represented number of samples, mean, and stand deviation of glaucoma patients and control subjects, respectively; ^c^ P and N represented the numbers of positive samples and the numbers of negative samples, respectively.

**Figure 3:**
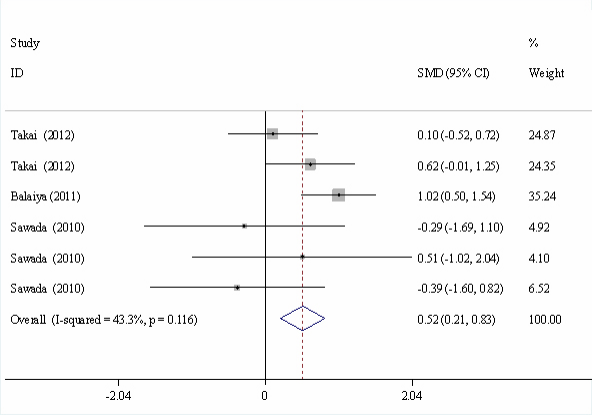
Forest plot of the standardized mean difference and 95% confidence intervals of the tumor necrosis factor alpha level in aqueous humor between the patients with open angle glaucoma and the control subjects. The black squares represent individual studies, and the size of the square represents the weight given to each study in the meta-analysis. The diamond represents the pooled estimate, and the horizontal line represents the 95% confidence interval. The pooled results showed that patients with open angle glaucoma had higher tumor necrosis factor alpha levels than the controls.

The results of the meta-analysis for the comparisons of TNF-α levels in AH between the patients with glaucoma and the controls are shown in Figure 3. The pooled results of six studies demonstrated that patients with OAG had higher TNF-α levels in AH than the control subjects (SMD=0.517, 95% CI=0.207–0.826, p=0.001). No significant heterogeneity was observed (*I*^2^=43.3%, p=0.116). Egger’s test and Begg’s funnel plot were applied for comparison to assess the publication bias of the literature, and no possibility of publication bias for this test was observed (p=0.191).

## Discussion

Glaucoma is the second leading cause of blindness worldwide. Growing evidence supports the important role of TNF-α as a mediator of neurodegeneration in OAG [3,5]. Some of these studies focused on the association between the *TNF-α* gene polymorphisms and OAG risks, in which several gene polymorphisms in the *TNF-α* gene promoter region including *TNF-α* (−308G/A, −238G/A, −863C/A, and −857C/T) have been investigated. However, these epidemiological studies obtained contradictory results, which might be related to factors such as the sample size of the study, the ethnicity of the enrolled subjects, and the type of OAG. Considering the potential important roles of TNF-α in the etiology of OAG, a timely meta-analysis was conducted to provide an appropriate approach to obtain a more definitive conclusion.

In the current study, 14, five, three, and three studies were included in the analyses for the associations between *TNF-α* −308G/A, −857C/T, −863C/A, and −238G/A polymorphisms and the risks of OAG, respectively. When all the studies were included, the combined results showed no significant association between the *TNF-α* −308G/A polymorphism and the risks of OAG. However, when the studies in which the genotype distribution in the control groups deviated from HWE were excluded [14,19], the pooled results clearly showed that the *TNF-α* −308G/A polymorphisms were significantly associated with the risks of HTG, but not with the risks of NTG or EXG. Furthermore, ethnicity-stratified subgroup analysis revealed that significant association also existed in Asians. Regarding the other genetic polymorphisms including −238G/A, −863C/A, and −857C/T, the results of the current meta-analysis demonstrated no significant association.

In a recently published meta-analysis, Yu et al. investigated the association between the *TNF-α* −308G/A polymorphism and the risks of glaucoma [33]. In that study, eight studies investigating the association between the *TNF-α* −308G/A polymorphism and the risks of glaucoma were included; those were included in the current study, in which five additional newly published studies were included [12,19,29]. Importantly, the meta-analysis by Yu et al. did not include subgroup analysis for HTG and NTG, which are apparently different, although both are types of POAG. HTG is characterized by high IOP, while the IOP of NTG is usually within normal levels. Findings of ongoing in vivo studies support that TNF-α and TNF-R1 are upregulated following experimental elevation of IOP, which indicates that a potential link may exist between TNF-α and IOP [5]. Furthermore, the only two studies in which the association between *TNF-α* −308G/A polymorphisms and the NTG risks was investigated reported null association [18,19]. Therefore, it is not surprising that the *TNF-α* −308G/A gene polymorphism was significantly associated with HTG risk, but not with NTG risks. EXG is another type of OAG with quite different characteristics from POAG. EXG is the most frequently reported type of secondary glaucoma, which is caused by deposition of exfoliation material and liberated iris pigment in the trabecular meshwork leading to elevation of IOP [34]. Thus, EXG may be significantly associated with other genetic polymorphisms such as the lysyl oxidase-like 1 (*LOXL1*) gene [35].

To investigate whether TNF-α might play important roles in the etiology of OAG, comparison of the TNF-α level between patients with OAG and controls would provide direct evidence. Therefore, in the present study, in addition to exploring the association between *TNF-α* gene polymorphisms and the risks of OAG, we summarized the studies in which the TNF-α levels in the AH were detected in patients with OAG as well as in control subjects. Some studies provided the mean and standard deviation of TNF*-*α levels in patients with OAG and controls, while other studies reported the positive and negative AH samples of patients with OAG and controls (Table 4). The pooled results of six individual studies showed that patients with OAG had higher TNF-α levels in AH than the control subjects (SMD=0.517, 95% CI=0.207–0.826, p=0.001; Figure 3). Other studies showed that patients with glaucoma were more likely to have detectable levels of TNF-α in their AH [21]. These data support the theory that TNF-α might play important roles in the etiology of OAG. However, most of these studies enrolled Japanese patients. Thus, whether similar results could be obtained in people in other countries remains unclear.

Despite the clear strengths of our study such as the larger sample size compared with the previous individual ones, it does have some limitations. First, the present meta-analysis was based primarily on unadjusted effect estimates and CIs for the association study between *TNF-α* gene polymorphisms and the OAG risks; thus, the effect estimates were relatively imprecise. Second, OAG is a multifactor disease, which may be related to genetic and environmental factors. However, the effects of gene–gene and gene–environment interactions were not addressed in this meta-analysis, and thus the potential roles of these gene polymorphisms may be masked or magnified by other gene–gene/gene–environment interactions. Third, although the funnel plot and Egger’s test showed no publication bias, selection bias may also exist because only published studies in the three selected databases were retrieved.

In summary, the current meta-analysis suggests that the *TNF-α* −308G/A gene polymorphism is significantly associated with the risks of HTG, but not with NTG or EXG, while the *TNF-α* (−238G/A, −863C/A, −857C/T) polymorphisms are not significantly associated with OAG risks. Furthermore, the pooled results of six individual studies indicate that patients with OAG may have higher TNF-α levels in AH than the control subjects, which supports the important roles of TNF-α in the development of OAG. Since potential confounders could not be ruled out completely, further studies are needed to confirm these results.
